# The Role of the Peyronie’s Disease Questionnaire in Translation, Cultural Adaptation, and Treatment Management for Portuguese-Speaking Populations

**DOI:** 10.7759/cureus.76242

**Published:** 2024-12-23

**Authors:** Ricardo H De Rizzo, Guilherme C Gonzales, Henrique R Cortines, Caiã C Fraga Carvalho, Leonardo De Rizzo, Mateus Henrique Silva Faria, Vinicius C Lopes, Fernando Nestor Facio Júnior, Luís Cesar Fava Spessoto, Andre C Pereira

**Affiliations:** 1 Department of Urology, Faculty of Medicine of São José do Rio Preto, São José do Rio Preto, BRA; 2 Department of Internal Medicine, Barão de Maua University Center, Ribeirão Preto, BRA

**Keywords:** cultural adaptation, patient-centered care, peyronie's disease, peyronie's disease questionnaire, therapeutic management

## Abstract

Peyronie's disease (PD) is characterized by the formation of fibrotic plaques within the penile connective tissue, leading to abnormal curvature, pain, and erectile dysfunction, profoundly affecting patients' physical and psychological well-being. The Peyronie's Disease Questionnaire (PDQ) is a validated instrument designed to assess key aspects of the disease, including pain, sexual function, and psychosocial impact. This narrative review underscores the importance of translating and culturally adapting the PDQ into Portuguese to enhance its applicability for Portuguese-speaking populations. The methodological process includes an analysis of the cultural adaptation of the PDQ and its impact on treatment. Articles from PubMed, Scopus, SciELO, and Google Scholar, published between 2000 and 2024, were reviewed. The study focused on the translation, validation, and cultural adaptation of patient-reported outcome measures (PROMs) for PD. Methodologies such as forward and backward translation, expert panel reviews, and psychometric validation were examined. The findings emphasize the importance of linguistic and cultural validation to improve the use of PROMs in Portuguese-speaking populations and enhance clinical management of the disease. The findings highlight the critical role of the PDQ in clinical management by enabling standardized assessments, personalized care, and optimized therapeutic strategies, ultimately improving outcomes for individuals with PD in diverse cultural settings.

## Introduction and background

Peyronie’s disease (PD) is a complex condition involving fibrous plaque formation within the penile tunica albuginea, causing abnormal curvature, erectile dysfunction, and pain during erection [1]. This pathological process can disrupt the normal elastic properties of the penile tissue, impairing its function and causing significant distress to affected individuals. Such physical and psychological burdens necessitate the development of robust tools for evaluating the disease's impact on patients’ lives, a gap addressed by instruments like the Peyronie’s Disease Questionnaire (PDQ) [2].

The PDQ is a validated patient-reported outcome measure (PROM) specifically designed to assess the multidimensional aspects of PD, including physical symptoms, psychological distress, and sexual function. It captures the subjective experience of patients through structured questions that encompass domains such as pain, penile curvature, and emotional well-being. Accurate translation and cultural adaptation of such tools are critical for their effective use across diverse linguistic and cultural populations. By aligning the PDQ with the cultural context of Portuguese-speaking patients, its applicability in clinical settings can be significantly enhanced, facilitating better communication between clinicians and patients [3].

The process of translating and culturally adapting the PDQ involves rigorous methodological steps to ensure its psychometric properties remain intact. Forward and backward translations, expert panel reviews, and pilot testing are integral to this process, enabling the instrument to retain its original intent while addressing cultural nuances. Similar methodologies have been successfully applied to other PROMs, such as the Satisfaction with Facial Appearance Overall (FACE-Q) and a PROM designed to evaluate outcomes for patients who are obese and undergo weight loss through diet, exercise and/or bariatric surgery/medicine, and body contouring patients (BODY-Q), underscoring the importance of adhering to established guidelines during translation and validation efforts [4-7]. The adaptation of the PDQ to Portuguese-speaking populations provides an opportunity to improve the management and treatment of PD in these regions [8].

A culturally adapted PDQ not only enhances clinical practice but also supports the standardization of patient data collection, which is crucial for advancing research in PD. Previous studies have demonstrated that translated PROMs can facilitate international collaborations and enable comparative analyses of patient outcomes across different populations. For instance, the Spanish, Danish, Italian, and French versions of the PDQ have shown how linguistic and cultural validation efforts contribute to broadening the instrument’s utility in diverse healthcare environments [8-11]. These findings underscore the transformative potential of adapting the PDQ into Portuguese to benefit both patients and healthcare providers.

The role of the PDQ in clinical management is paramount, as it provides a structured framework for evaluating treatment outcomes and understanding the broader impact of PD on patients’ quality of life. By addressing the specific needs of Portuguese-speaking patients, the adapted PDQ can inform more personalized and effective treatment strategies. This aligns with global trends in patient-centered care, emphasizing the importance of incorporating validated PROMs into routine clinical practice to enhance decision-making and patient satisfaction [12].

In conclusion, the adaptation of the PDQ into Portuguese represents a pivotal advancement in the management of PD for Portuguese-speaking populations. This study aims to explore the translation and cultural adaptation process, highlighting its significance for improving patient outcomes and fostering international research collaborations. By leveraging established methodologies and incorporating cultural considerations, the adapted PDQ has the potential to transform clinical practice, ensuring that patients with PD receive the highest standard of care tailored to their unique cultural and linguistic contexts [2,3,10,13].

## Review

Methods

This study employed a narrative review to analyze the translation and cultural adaptation processes of the PDQ and its impact on the treatment of the disease. Articles were sourced from various scientific databases, including PubMed, Scopus, SciELO, and Google Scholar, to ensure a comprehensive exploration of the topic. These platforms were selected for their reliability and extensive coverage of medical, psychological, and methodological research. The review included studies published in peer-reviewed journals, focusing on cross-cultural adaptation, linguistic validation, and the application of PROMs [3,14]. Studies in English published between 2000 and 2024 were selected. One study in Portuguese was included because outlines its provides a structured checklist for the translation and cross-cultural adaptation of health questionnaires and outlines clear steps to ensure semantic, cultural, and conceptual equivalence between the original and adapted versions [14]. Search terms included “Peyronie’s Disease,” “Peyronie’s Disease Questionnaire,” “translation,” and “cultural adaptation.”

The inclusion criteria for the articles were predefined to ensure relevance and quality. Studies addressing the translation and validation of PROMs, particularly those demonstrating their impact on the treatment of PD or similar conditions, were selected. Articles with clear methodologies for cross-cultural adaptations were prioritized as they directly supported the goals of this research. Non-peer-reviewed publications and studies lacking detailed methodology or unrelated to PROMs were excluded. This rigorous selection ensured the dataset comprised high-quality studies relevant to the research objectives.

Data extraction focused on identifying key themes, methodologies, and findings related to the translation and adaptation of the PDQ. Particular attention was given to linguistic validation steps, such as forward and backward translations, expert panel reviews, pilot testing, and test-retest reliability. Additionally, the studies were analyzed for their approaches to cultural equivalence, psychometric validation, and practical application in clinical settings. These elements were essential for aligning the PDQ with the cultural and linguistic needs of Portuguese-speaking populations.

The review process emphasized methodological rigor, adhering to established guidelines for reviews and cross-cultural adaptation. Selected articles were critically appraised for validity, reliability, and relevance. These practices ensured the findings were based on robust evidence, enhancing the study's academic credibility.

Finally, the data synthesis provided a comprehensive understanding of challenges and best practices in translating and adapting the PDQ. The findings underscore the importance of linguistic and cultural validation in improving the applicability of PROMs across diverse populations. This methodological approach not only facilitated a comprehensive literature review but also provided insights into advancing the clinical management of PD in Portuguese-speaking regions.

Steps for cross-cultural adaptation of self-reported instruments

**Figure 1:**
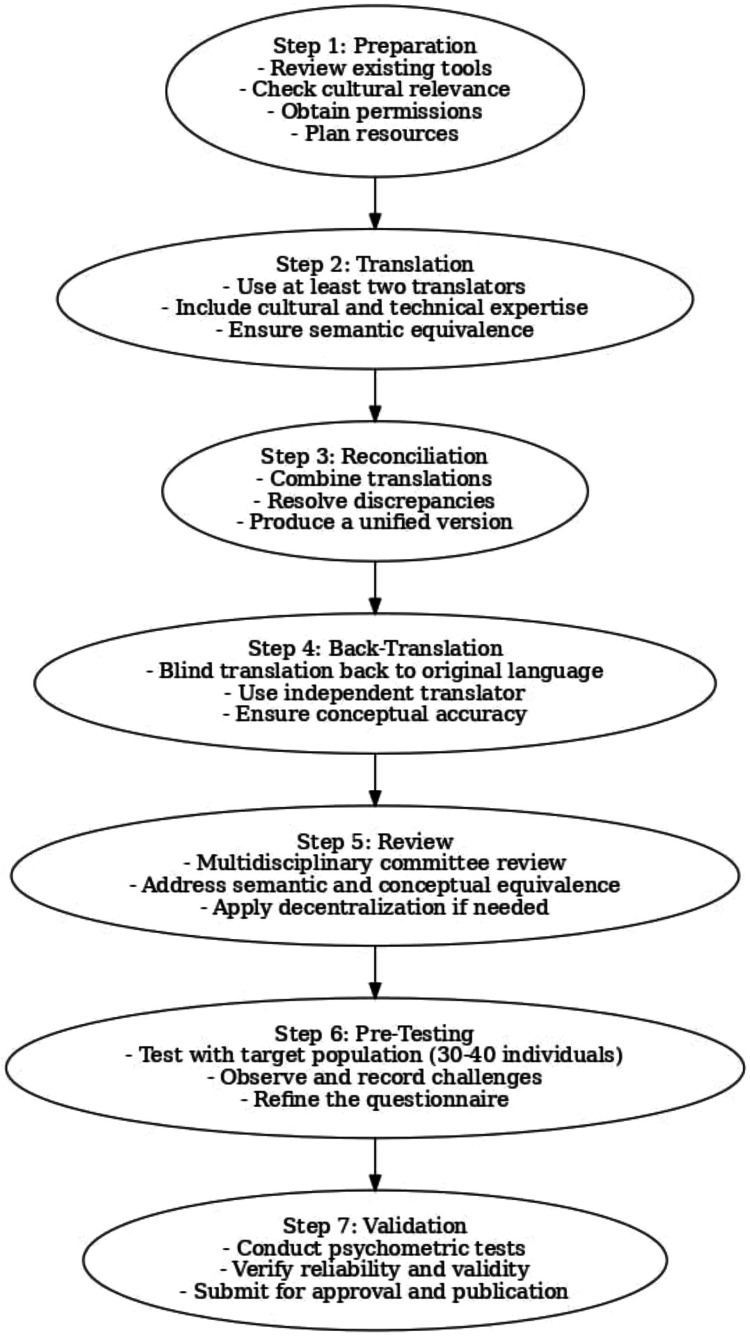
Steps for cultural adaptation of questionnaires and assessment tools Based on the content of Fortes and Prufer (2019) [14]. Licensed under CC BY 4.0.

The process of cross-cultural adaptation of self-report measures is essential to ensure equivalence between different languages and cultures. The methodology aims to achieve semantic, idiomatic, experiential, and conceptual equivalence while maintaining the original content's validity. The process follows seven key steps as shown in Figure 1 [14].

Initially, the preparation phase ensures the feasibility of the cultural adaptation process. Key activities include reviewing existing tools, confirming conceptual equivalence with the target culture, obtaining permission from the instrument's original authors, and ensuring the availability of resources. After that, two independent translations are produced from the source language to the target language by bilingual translators. One translator is knowledgeable about the questionnaire's concepts, while the other is "naive" and unfamiliar with it. Discrepancies between translations are discussed and resolved. Next, the two translations are synthesized into a single version, accompanied by a detailed report documenting how any issues were addressed. The synthesized version is then back-translated into the source language by translators who are blind to the original questionnaire, ensuring content fidelity and helping to identify inconsistencies or errors.

Subsequently, a multidisciplinary expert committee, including methodologists, healthcare professionals, linguists, and translators, reviews all versions to evaluate semantic, idiomatic, experiential, and conceptual equivalence, ultimately producing a pre-final version of the questionnaire. This pre-final version is then tested with 30-40 participants from the target population to identify potential issues related to item interpretation and response patterns, ensuring equivalence in practical application. Finally, all reports and documentation are submitted to the questionnaire developers or a coordinating committee to verify that the process was correctly followed. After adaptation, further testing of psychometric properties such as validity and reliability is recommended to ensure the measure's robustness in the new cultural context [3,14].

Pathophysiology of PD

PD is characterized by the formation of fibrous plaques within the tunica albuginea of the penis, leading to deformity, pain, and potential erectile dysfunction. This condition typically results from repetitive microtrauma during intercourse or other penile manipulations, causing aberrant wound healing. The process involves an imbalance between collagen deposition and degradation, where an excess of type I collagen and a reduction in type III collagen contribute to plaque formation. Additionally, myofibroblasts play a significant role by promoting fibrosis and perpetuating plaque rigidity [2,12,15]. The chronic inflammatory response further exacerbates tissue remodeling, underlining the need for a better understanding of its molecular underpinnings.

The pathophysiological progression of PD begins with localized vascular injury, leading to inflammation and subsequent fibroblast activation. Transforming growth factor-beta (TGF-β) and other pro-fibrotic cytokines are upregulated, promoting extracellular matrix deposition and tissue fibrosis [16]. Overexpression of oxidative stress markers and reduced nitric oxide availability have also been implicated in disease progression. These factors collectively contribute to impaired penile elasticity and curvature, which often result in compromised sexual function [10,15]. Furthermore, genetic predisposition may influence susceptibility to PD, as polymorphisms in genes regulating wound healing and fibrosis have been observed in affected individuals [8].

Advanced stages of PD are marked by calcification within plaques, which significantly reduces treatment efficacy and complicates surgical interventions. Plaque calcification is thought to stem from chronic inflammation and prolonged exposure to pro-fibrotic stimuli, further aggravating penile rigidity and deformity. Studies suggest that calcium deposition correlates with increased patient morbidity, including worsening pain and psychological distress [12,17,18]. Psychosexual effects, such as reduced self-esteem and strained interpersonal relationships, are prevalent among patients, necessitating a holistic approach to management [19,20].

Contemporary research highlights the importance of early diagnosis and intervention to mitigate disease progression. The use of PROMs, such as the PDQ, has facilitated a better understanding of the disease’s impact on quality of life and treatment outcomes [3,13]. Non-surgical treatments, including intralesional injections of collagenase *clostridium histolyticum*, aim to disrupt plaque structure and restore penile function. However, these therapies are most effective during the acute phase, when inflammation and fibrosis are still modifiable [21,22].

Ongoing studies focus on elucidating the molecular mechanisms underlying PD to identify novel therapeutic targets. Gene editing tools, anti-fibrotic agents, and advanced imaging techniques represent promising avenues for future research [17]. Additionally, cross-cultural adaptations of diagnostic tools enhance the global understanding of PD, enabling better patient care across diverse populations [2,8]. A multidisciplinary approach integrating medical, psychological, and surgical expertise is crucial to addressing the complex challenges posed by this debilitating condition [22].

Insights and structure of the PDQ

The PDQ is a comprehensive, 15-item, self-administered, disease-specific, and multidimensional instrument designed to assess the symptoms, psychosocial impact, and functional limitations associated with PD, a condition characterized by the development of fibrous scar tissue inside the penis, leading to deformity and pain [13]. The PDQ addresses a broad range of concerns that patients with PD may experience, including anxiety about potential damage to the penis, concerns about penile bending or collapsing, difficulties with vaginal penetration, discomfort during intercourse, and limitations in sexual positions. The questionnaire provides valuable insights into both the physical and psychological burdens of the disease, which may affect the overall quality of life.

The instrument is structured into three distinct subscales: the "psychological and physical symptoms" subscale (comprising six items), the "penile pain" subscale (three items), and the "symptom bother" subscale (four scored items and two yes/no questions). These subscales collectively capture the full spectrum of the impact of PD, from its physical manifestations to its psychological consequences. The "psychological and physical symptoms" domain employs a 5-point Likert scale that ranges from "None" to "Very Severe," enabling patients to rate the severity of symptoms they experience. In the "penile pain" domain, patients are asked to evaluate the intensity of pain they have experienced over the past 24 hours, with pain severity rated on a numeric scale from 0 to 10. This helps to quantify the daily pain levels and monitor changes over time. The "symptom bother" domain assesses the level of distress caused by symptoms using a 5-point Likert scale, ranging from "not at all bothered" to "extremely bothered," allowing for a nuanced understanding of how much the symptoms interfere with daily life and sexual functioning [23].

Each domain of the PDQ is scored separately, as the instrument does not produce a total score. This separation of domains allows for a more detailed and specific analysis of the various aspects of the disease's impact. Higher scores within each domain indicate a greater negative impact on the patient’s physical and emotional well-being. Specifically, the score range for the "psychological and physical symptoms" domain is from 0 to 24, for the "penile pain" domain from 0 to 30, and for the "symptom bother" domain from 0 to 16 [23]. These scores provide a quantitative measure of the severity of symptoms, which can guide clinical decisions and patient management. The PDQ is particularly valuable in clinical practice and research, offering a reliable tool for assessing treatment outcomes and the effectiveness of therapeutic interventions.

Relevance of the PDQ in the diagnosis and evaluation of patients

The PDQ has become an essential tool in evaluating and diagnosing PD, providing valuable insights into the patient's condition. This instrument assesses both the physical and psychological impacts of the disease, offering healthcare providers a comprehensive understanding of its effects. The significance of such questionnaires lies in their ability to highlight the often-underreported emotional and physical challenges faced by patients due to the sensitive nature of the condition. Several studies have emphasized the importance of PROMs in improving diagnosis and treatment strategies [12,24]. By integrating these patient perspectives into the clinical practice, healthcare providers can develop more tailored and effective treatment plans.

A key aspect of the PDQ's utility is its cultural adaptability. To ensure accurate and reliable data collection, the questionnaire must be appropriately translated and validated in different languages. This process requires careful preservation of the original questionnaire's meaning while considering cultural nuances. Research has shown that proper adaptation of these tools can enhance their effectiveness in diverse populations [3]. The linguistic validation process ensures that the instrument is both comprehensible and applicable to local populations, which is essential for the reliability of the data obtained [10].

Additionally, the PDQ has proven instrumental in tracking changes in a patient's condition over time. This longitudinal assessment is vital for understanding how different interventions affect both the physical symptoms and the emotional well-being of the patients. The ability to monitor disease progression and the impact of various treatments on patients' quality of life helps clinicians refine therapeutic approaches, improving both patient satisfaction and clinical outcomes [2]. This underscores the utility of the PDQ in not only diagnosis but also in therapeutic management.

In clinical practice, the PDQ fosters communication between healthcare providers and patients, especially in cases where discussing the symptoms of PD may be uncomfortable. By using a standardized tool like the PDQ, clinicians can initiate conversations with patients more effectively and with greater sensitivity. This approach facilitates a more open dialogue, which can lead to a more accurate assessment of the patient’s condition and a better understanding of their concerns and expectations regarding treatment options [8]. This also reinforces the patient-centered care approach, which is increasingly emphasized in modern healthcare.

The PDQ's role in cross-cultural adaptation is paramount, ensuring its effectiveness in diverse settings. To maintain validity in different languages, the translation process must account not only for linguistic differences but also for cultural attitudes towards the disease, which can vary significantly across regions. As seen in various international studies, such cultural nuances are integral to the success of the tool [8-11]. Therefore, the accuracy of the PDQ’s findings depends heavily on its cultural relevance, which requires careful attention to local beliefs and practices.

Another critical aspect of the PDQs is its ability to evaluate the psychological impact of PD. Many patients experience feelings of embarrassment, anxiety, and depression due to the disease's physical manifestations. By incorporating these emotional factors into the diagnostic process, the PDQ provides a holistic view of the patient's health status [25]. This comprehensive approach not only diagnostic accuracy but also facilitates more effective treatment planning, addressing the full spectrum of issues that patients with PD face [1,2].

Beyond its clinical utility, the PDQ serves as a valuable research tool, gathering data that can guide future studies on PD. This evidence-based approach has significantly contributed to the development of more effective treatment protocols. Its use in clinical trials has deepened the understanding of how various treatments impact both the physical and psychological dimensions of the disease [26]. Thus, the PDQ plays a pivotal in advancing medical research and improving therapeutic approaches.

Finally, the importance of the PDQ lies in its contribution to the overall management of PD, promoting both physical and emotional well-being. By enabling the development of personalized treatment strategies tailored to the unique needs of each patient, the tools underscore its enduring value. Its continued use and adaptation in clinical practice are vital for improving the quality of life for those suffering from PD, reinforcing its importance in both clinical and research settings [13].

Contributions of the PDQ to the management and treatment of PD

PD, characterized by penile curvature due to fibrosis of the tunica albuginea, poses significant challenges not only in terms of physical symptoms but also psychological well-being. The PDQ has become an essential tool for assessing and managing this condition. By providing a structured method to evaluate the severity of symptoms, it allows healthcare providers to better understand the patient’s experience. The importance of PROMs in the management of PD is evident in various studies, emphasizing the role of tools like the PDQ in delivering personalized and effective treatment approaches [10]. This is crucial in a condition where emotional and functional impacts are as significant as physical ones.

A key contribution of the PDQ lies in its adaptability across different cultural and linguistic contexts. As highlighted by various authors, the PDQ’s validity and reliability across different linguistic and cultural settings ensure that the data gathered reflects the true patient experience in diverse populations. For instance, the translation of the PDQ into multiple languages, including Spanish, Danish, Italian, and French, has enhanced its global applicability, making it a useful tool for both clinicians and researchers worldwide [8-11]. This process of cultural adaptation is fundamental to ensuring that the instrument accurately captures the nuances of how PD affects individuals from different backgrounds.

In addition, the PDQ plays a critical role in guiding treatment decisions by addressing both the physical and psychological components of PD [24]. Since the condition impacts not only penile deformity but also sexual function and quality of life, the questionnaire identifies specific areas of concern. This comprehensive assessment supports tailored treatment strategies, ranging from pharmacological interventions to surgical options, while ensuring that psychological impacts are also addressed [2,26]. As a result, PDQ contributes significantly to patient-centered care, aligning treatment decisions with individual priorities and needs.

Furthermore, the PDQ is invaluable for monitoring treatment outcomes over time. It facilitates longitudinal evaluations of symptom progression or improvement, offering valuable insights into the effectiveness of various treatments. This is particularly useful in clinical trials and real-world settings where treatment modalities may vary, but the need for robust outcome measures remains constant. The ability to track changes in the severity of symptoms through validated questionnaires like the PDQ ensures that healthcare providers can make informed decisions regarding treatment adjustments or transitions [12].

In conclusion, the PDQ has significantly contributed to the management and treatment of PD. It enhances the clinical understanding of the condition, supports cultural adaptability, guides treatment decisions, and provides a reliable means of assessing treatment outcomes. As further studies validate its effectiveness across different settings and populations, the questionnaire remains a cornerstone in both clinical practice and research related to PD [3,13,27]. Its ongoing use holds promise for improving patient care worldwide.

Challenges in translation and cultural adaptation

The process of translating and adapting questionnaires for cross-cultural use is essential to ensuring that research tools maintain validity and reliability across different populations. These adaptations go beyond mere linguistic translation, addressing cultural differences that may influence how items and responses are interpreted. Effective adaptation ensures that the tool remains relevant and accurate for measuring the same constructs in diverse cultural contexts. For instance, the PDQ has undergone several translations and cultural adaptations to ensure its applicability in various languages and regions, highlighting the need for a thorough methodological approach to such processes [8,10].

Cultural adaptation often involves modifying the content of a questionnaire to align with local cultural norms and practices while preserving its original intent and construct. Guidelines for cross-cultural adaptation emphasize the importance of not only translating the words but also adapting idiomatic expressions and culturally specific concepts to ensure the tool resonates with respondents in different regions [3]. This step is crucial for preserving the integrity and relevance of the instrument, particularly in medical and psychological assessments where cultural nuances can significantly affect participant responses.

Moreover, the process of validation plays a pivotal role in ensuring that the adapted questionnaire produces reliable and valid results when used in a new cultural setting. Validation involves testing the psychometric properties of the adapted tool, such as its reliability, validity, and sensitivity to cultural differences. In the case of the PDQ, studies have validated its use in languages such as Spanish, Danish, and Italian, confirming that the adapted versions maintain the tool’s effectiveness in assessing the condition across various cultural contexts [8,10,11]. This ensures that the translated questionnaire is not only linguistically accurate but also culturally meaningful and scientifically rigorous.

One of the critical challenges in the cultural adaptation process is ensuring that the adapted questionnaire is sensitive to the cultural and psychological contexts of the target population. For example, an article discusses how cultural perceptions of health conditions, such as PD, can differ significantly between countries, affecting how patients perceive their symptoms and how they respond to health-related questions [2]. Therefore, a culturally adapted questionnaire must be flexible enough to capture these differences while still providing consistent and comparable data across different groups.

It is essential to recognize that the process of cross-cultural adaptation is not static but requires ongoing evaluation and adjustment. As global health trends evolve and cultural perspectives change, it is crucial to revisit and revise adaptation protocols to maintain their relevance. Continuous improvement of tools like the PDQ ensures they remain valuable resources for clinical research and patient care, offering reliable insights into conditions like PD, which can significantly impact patients’ quality of life [12].

However, certain limitations must be acknowledged. The scarcity of robust epidemiological data on PD in Portuguese-speaking regions may hinder the generalizability of findings across diverse settings. Additionally, cultural stigmas related to sexual health could affect patient engagement with the questionnaire, potentially limiting its widespread adoption. Future studies should focus on validating the PDQ in real-world clinical settings, exploring its applicability in diverse patient groups, and addressing these cultural and logistical challenges.

## Conclusions

The PDQ remains a vital tool for advancing both clinical practice and research, paving the way for more inclusive and effective management strategies tailored to Portuguese-speaking patients. The translation and cultural adaptation of the PDQ into Portuguese represent a significant advancement in the management of PD for Portuguese-speaking populations. By ensuring linguistic accuracy and cultural relevance, the adapted PDQ provides clinicians with a powerful tool to assess the multifaceted impacts of the disease, encompassing physical, psychological, and social dimensions. This effort supports improved diagnosis, personalized treatment plans, and enhanced communication between healthcare providers and patients, fostering better clinical outcomes and patient satisfaction.
